# Determination of Soil Cadmium Safety Thresholds for Food Production in a Rice-Crayfish Coculture System

**DOI:** 10.3390/foods11233828

**Published:** 2022-11-27

**Authors:** Hui Gao, Xiang Peng, Linxiu Dai, Jingyong Li, Qian Yang, Zhi Dou, Qiang Xu

**Affiliations:** 1 Jiangsu Key Laboratory of Crop Genetics and Physiology, Jiangsu Key Laboratory of Crop Cultivation and Physiology, Agricultural College of Yangzhou University, Yangzhou 225009, China; 2 Jiangsu Co-Innovation Center for Modern Production Technology of Grain Crops, Yangzhou University, Yangzhou 225009, China; 3 Research Institute of Rice Industrial Engineering Technology of Yangzhou University, Yangzhou 225009, China

**Keywords:** rice-crayfish coculture, cadmium, heavy metals, safety threshold value, food safety, bioaccumulation

## Abstract

Previous studies have mainly focused on cadmium (Cd) contamination in conventional rice monocultures, and no research on rice-crayfish coculture has been reported. In this study, a Cd-contaminated (0–30 mg kg^−1^) rice-crayfish co-culture system was established by adding exogenous Cd. The results showed that the Cd concentration in each tissue of rice and each organ of crayfish increased with increasing soil Cd concentration. Specifically, the Cd concentration in each rice tissue was as follows: root > stem > leaf ≈ panicle > grain > brown rice, and the jointing and heading stages were critical periods for the rapid enrichment of Cd in the aboveground tissues of rice. The Cd concentration in each organ of crayfish was as follows: hepatopancreas > gut > gill ≈ exoskeleton > abdominal muscle. Cd was gradually enriched in the abdominal muscle after 30 days of coculture between crayfish and rice. Pearson’s correlation analysis showed that the soil’s total Cd concentration, available Cd concentration, and water Cd concentration were positively correlated with Cd content in various tissues of rice and various organs of crayfish, whereas EC and TDS in water were markedly related to rice stems, leaves, stalks, and small crayfish. According to the maximum limit of Cd in grain (0.2 mg kg^−1^) and crustacean aquatic products (0.5 mg kg^−1^) in China, the safe threshold of soil Cd for rice and crayfish under the rice-crayfish coculture system is 3.67 and 14.62 mg kg^−1^, respectively. Therefore, when the soil Cd concentration in the rice-crayfish coculture system exceeds 3.67 mg kg^−1^, the safety risk to humans through the consumption of food from this coculture system will increase. This study provides a theoretical basis for safe food production in a rice-crayfish coculture system using the established Cd pollution model.

## 1. Introduction

Economic development and the acceleration of urbanization and industrialization have led to an increase in heavy metal pollution in soils. According to the “National Soil Pollution Survey Bulletin” released in 2014, approximately 20 million hectares of arable land in China has been polluted by heavy metals to varying degrees, of which nearly 3.33 million ha is heavily and moderately polluted. Most of this land is located in economically developed areas [1]. Application of sewage sludge, farmyard manure, or some commercial fertilizers is the main source of Cd in the agriculture soil [2,3]. Cadmium (Cd) is one of the most toxic trace heavy metals to plants and animals. Compared with other trace elements, Cd in soil is more mobile and is more likely to be bioaccumulated in the food chain [4,5], thus endangering the lives of humans and animals. The daily diet accounts for 90% of Cd in the human body, and the half-life of Cd in the human body is 15–20 a. Therefore, long-term consumption of foods with slightly excessive Cd will lead to the accumulation of Cd in the human body and cause chronic toxicity, causing diseases such as cancer, arthritis, emphysema, and renal tubular necrosis [6,7].

Integrated farming of rice and aquatic animals (IFRAA) is an ecological agriculture model that combines rice cultivation and aquatic animal aquaculture with field engineering to obtain higher economic benefits [8]. The IFRAA is widely distributed in Asian countries, such as China, Bangladesh, Myanmar, and Vietnam [9]. In recent years, China’s IFRAA has rapidly expanded [10,11], owing to the strong demand for high-economic-value aquatic products (such as crayfish, shrimp, crab, and fish) [12] and the increasing shortage of arable land resources. This is against the background of China’s agricultural supply side structural reform. By 2020, the total area of IFRAA in China reached 2.33 million ha, of which the total area of rice-crayfish coculture was 1.26 million ha, accounting for the largest proportion of 47.7%, and it is still expanding rapidly [13,14]. The rice-crayfish coculture can produce carbohydrates and aquatic animal protein using limited water and soil resources, reduce the application of chemical fertilizers and pesticides [15], improve the physical and chemical properties of soil [16], and promote the energy cycle and efficient utilization of material resources in the agricultural system [17,18], which is important for ensuring agri-food safety. The sustainable development of the ecological environment is of great significance.

With the intensification of heavy metal pollution and the rapid expansion of rice-crayfish coculture, the risk of human exposure to paddy field pollutants through the consumption of rice and crayfish increases. This is achieved through arable soil, irrigation water, pesticides, organic and chemical fertilizers, and aquatic animal feed [19]. Rice is a staple food for nearly half of the world’s population, especially in Asia [20]. Unfortunately, populations on a rice-based diet are more susceptible to Cd, as rice grown in Cd-contaminated soils are particularly prone to Cd accumulation [21]. Rice consumption is the dominant source of Cd intake by the Asian population. For example, it accounts for 65% and 77% of the total Cd intake for urban and rural residents in southern China, respectively [22]. In addition, crayfish are widely favored because of their low price and delicious taste. Crayfish possess high nutrient levels in aquatic ecosystems and can accumulate Cd from water and consume Cd-contaminated food. The concentration of heavy metal pollutants in their muscles can be several times higher than that in the surrounding environment [23,24], which threatens human safety and health through the amplification and transmission of the food chain. Although there are many studies on the migration, transformation, and safety risks of the heavy metal Cd in rice fields or aquaculture water [25,26], studies on Cd contamination in the rice-crayfish coculture are very rare, and the soil and water environments of the coculture system are very different from those of traditional paddy fields [27]. The transformation, migration, and cycling of Cd in the rice-crayfish coculture occurred at the rice-crayfish-soil-water interface. The distribution pattern and cycling characteristics of Cd in rice-crayfish-soil-water may be affected by Cd concentrations and their chemical forms in the soil and water, systematic Cd input, soil disturbance by crayfish, and the Cd enrichment degree of rice. To the best of our knowledge, only one study has reported the distribution of Cd in crayfish tissues under a Chinese rice-crayfish coculture and performed a human health risk assessment of Cd [28]. Their research, to some extent, has broadened our understanding of Cd content in crayfish under rice-crayfish coculture. However, due to the limitations of investigation methods and the lack of continuous monitoring of Cd content in rice (or crayfish) and environmental factors, their study could not provide more information on Cd migration and transformation under the rice-crayfish coculture and the relationship between Cd levels of the research objects and environmental factors.

In this study, we used an established Cd pollution model under a rice-crayfish coculture and attempted to reveal the migration and transformation characteristics of Cd in the rice-crayfish coculture system through continuous dynamic monitoring of Cd at the water-soil-rice-crayfish interface. The specific objectives were: (1) to clarify the enrichment characteristics of Cd in various tissues and organs of rice and crayfish under the rice-crayfish coculture mode; (2) to explore the relationships between environmental (water and soil) factors and Cd content in rice and crayfish; (3) to determine the soil Cd safety threshold value under the rice-crayfish coculture mode.

## 2. Materials and Methods

### 2.1. Study Area

The study site (Figure S1) is in Xuyi County, Huai’an City, Jiangsu Province (118°40′17″ E, 32°59′55″ N), located in the transition area between the northern subtropical zone and the warm temperate zone, with a humid monsoon climate, abundant sunlight, and abundant rainfall. The annual average sunshine hours were 2222 h, the annual average temperature was 14.7 °C, the frost-free period was 215 days, and the annual average rainfall was 1.005 mm.

### 2.2. Experimental Design

#### 2.2.1. Establishment of Cd-Contaminated RCC Ecosystem

Soil samples for the experiment were collected from Gaoqiao Town, Xuyi County, at a depth of 0–0.4 m. After removing impurities manually, they were air-dried for one week and passed through a 10-mesh sieve. The soil type was waterlogged paddy soil with a pH of 7.8 and a soil organic matter content of 30.2 g kg^−1^. The background concentration of Cd in soil was 0.60 mg kg^−1^.

In 2021, a rectangular canvas pool was reinforced with steel bars to artificially establish a rice-crayfish co-culture ecosystem. The length, width, and height of the pool were 4.0 × 2.5 × 1.0 m. A total of 1500 kg of soil was added to each experimental pool. When soil was added, the bottom of the pool was close to the ground to ensure that it was flat, and the experimental pools were adjacent to each other. The soil was filled into the pool in a step-by-step method of adding soil in 15 rounds; that is, after each round of adding 100 kg of soil to each pool, the next round was added. Cadmium (CdCl_2_) (Shanghai Macklin Biochemical Co., Ltd., Shanghai, China) was added to the soil, and five treatments were set at 0, 1, 5, 15, and 30 mg kg^−1^, namely T0, T1, T2, T3, and T4, respectively, and each treatment was repeated three times. The cadmium reagent was added at the same time when the soil was put into the pool, and the cadmium reagent to be added in each pool was equally divided into 15 parts, each of which was dissolved in 5 L of water and added to the soil in rounds after it was completely dissolved. After every 100 kg of soil was added, cadmium solution was sprayed. To avoid the interference of rainwater in the experiment, a shed was built above the pool and covered with a light-transmitting shed film that could be retracted at any time according to the rainfall.

#### 2.2.2. Management of Experimental Plot

The rice variety was Nanjing 5718 which has good lodging resistance. It was sown on 28 May and transplanted manually on 26 June. The planting density was 30.0 × 12.4 cm with three seedlings per hill, and the basic seedling is 806,000 ha^−1^. The N fertilizer used was controlled-release urea with an N content of 46%. The controlled-release period was 80 d, and the total N application rate was 240 kg N ha^−1^. The P and K fertilizers used were calcium superphosphate and potassium chloride, with application rates of 150 kg P_2_O_5_ ha^−1^ and 150 kg K_2_O ha^−1^, respectively. A one-time basal application was used for all fertilizers. All the fertilizers used in this study were purchased from Anhui Maoshi Agricultural Technology Co., Ltd. (Heifei City, China). Deep well water was used for irrigation during the experiment, and no cadmium was detected. After the soil was completely placed into the pool, water was stored at a height of 3–5 cm from the soil surface, and a rake was used to flatten the soil surface. The rice was irrigated with shallow water (3–5 cm) from the transplanting stage to the jointing stage, and water was gradually added ~25 cm from the soil layer after the crayfish was placed in the pool at the heading stage. During the experiment, the high temperature caused water in the pool to evaporate. Thus, the water level was checked and replenished 3 days before each sampling.

The experimental crayfish (*Procambarus clarkii*) was purchased from Jiangsu Xuyi Crayfish Industry Development Co., Ltd. (Xuyi County, Huai’an City, China). Crayfish larvae with a clean body, healthy and disease-free status, strong vitality, and consistent specifications were selected as experimental materials. The crayfish larvae had an average weight of 10 g ind.^−1^. The stocking density was 6 ind. m^–2^, and a total of ~60 ind. were placed in each pool of T1, T2, T3, and T4 treatments. It should be noted that, in general, for the rice-crayfish coculture mode in Xuyi County, crayfish would be introduced into paddy fields after the jointing stage. However, to prevent the death of crayfish due to the high temperature during the experimental period in 2021, the time of this experiment was postponed to the heading stage (29 August). Crayfish was introduced on 27 October, and the growth period lasted for 59 days. The feed was provided on time at 18:00 every day, according to 4% of the crayfish body weight [29,30]. The feed was purchased from Tongwei Co., Ltd. (Chengdu City, China). Owing to the small change in the total body weight of crayfish during the experimental period, the daily feed amount remained unchanged until the day before sampling, and the total feed amount of each pool was 1.89 kg. The feed used is from Chia Tai Feed Co., Ltd., Hangzhou, China, and the Cd content in the feed is determined to be 0.14 mg kg^−1^.

### 2.3. Sample Collection and Measurements

#### 2.3.1. Sampling Frequency

Specific sampling arrangements are listed in Supplementary Table S1. Water and soil samples were collected every 7–10 days and mixed into one sample using a 5-point random sampling method. The rice samples were collected at different stages of growth. The samples at the tillering and jointing stages were divided into root, stem, and leaf. The samples at the heading stage, 20 days after the heading stage, and the mature stage were divided into root, stem, leaf, and panicle. The mature samples were divided into rice grain and brown rice. For crayfish samples, five crayfish from each pool were harvested in ground cages every 7 days. The crayfish collected were quickly frozen in liquid nitrogen and dissected into the hepatopancreas, gut, gill, external skeleton, and abdominal muscles [31]. The same parts of five crayfish were mixed into one sample and then stored in a sealing bag at –20 °C for later measurements.

#### 2.3.2. In Situ Determination of Physicochemical Indicators of Water and Soil

Physicochemical indicators of water and soil in each pool were measured in situ every 7–10 days. Water pH, dissolved oxygen (DO), and temperature were measured using a multi-parameter water quality detector (YSI Prosolo, COLO, Yellow Springs, OH, USA), water turbidity was measured with a portable turbidimeter (Hach 2100 Q, OSU, Loveland, CO, USA), water conductivity and TDS were measured with a portable TDS meter (Hengxin AZ8302, Taiwan, China), and soil ORP and pH were measured with a portable redox potential meter (Orion Star A221, Waltham, MA, USA).

#### 2.3.3. Determination of Cd

The collected water samples were mixed and divided into two bottles, each of which was placed in a 150 mL polyethylene bottle for the determination of total Cd (Cd_T_) and dissolved Cd (Cd_D_). The water samples were filtered with an ordinary filter to measure the T_Cd_ and filtered through 0.45 μm membrane filters to measure the D_Cd_. Filtered and acid treated samples measuring 100 mL were heated with 5 mL concentrated HNO_3_ on a hot plate at 95 °C and reduced to a final volume of 10 mL. The concentrations of T_Cd_ and D_Cd_ in the water were determined by inductively coupled plasma atomic emission spectroscopy (ICP-AES, Thermo 6300, Waltham, MA, USA).

The air-dried soil samples were ground with a mortar and pestle and passed through a 100-mesh sieve for the determination of Cd_T_ and available cadmium (Cd_A_). To the 0.25 g soil sample, 6 mL of HNO_3_, 3 mL of HCL, and 2 mL of HF were added in turns and digested with a microwave digestion system. After digestion was complete, the digested solution was placed in an electric heating furnace to remove the acid. Soil A_Cd_ was extracted using the DTPA-TEA method, and the extract was filtered through a 0.45 μm microporous membrane. T_Cd_ and A_Cd_ content in soil was determined by ICP-AES.

A half gram (dry weight) of rice tissue sample passed through a 45-mesh sieve were put into a microwave digestion tank, and 5 mL HNO_3_ and 2 mL of H_2_O_2_ were added for digestion. The digested solution, which was colorless or light yellow, was further heated to drive away acid, transferred to a 50 mL volumetric flask, and the T_Cd_ content was determined by ICP-AES.

One gram (wet weight) of crayfish tissue sample, a few glass beads, and 10 mL (9 + 1) of a mixed solution of HNO_3_ and HClO_4_ were placed into a conical flask for digestion overnight. The next day, the conical flask was placed on an electric hot plate for digestion until the digested solution was colorless or slightly yellow, after which the solution was used to determine the T_Cd_ content.

### 2.4. Harvest of Crayfish and Rice

The crayfish was harvested on October 26. After being caught in ground cages, the water was dried with a qualitative filter paper and weighed. The rice was harvested on 29 October. All plants from a 4.75 m^2^ area (120 hills) in each plot were harvested to determine grain yield and adjusted to 14% moisture content. Plants from 1.19 m^2^ (30 hills) in each plot were collected to determine the number of spikelets per panicle, filled grain percentage, and 1000-grain weight.

### 2.5. Statistical Analysis

All data are expressed as the mean ± standard error. The differences in the physicochemical indicators of water and soil at different growth periods were analyzed using variance analysis with the IBM SPSS 26 statistical software program (SPSS Inc., Chicago, IL, USA). The differences in Cd concentrations in various tissues of rice or crayfish among different treatments were analyzed using variance analysis. Pearson’s correlation analysis was used to analyze the relationship between Cd content in rice tissues and crayfish organs and environmental factors, water, and soil, and the correlation analysis heatmap was drawn using Origin 2021 software (OriginLab Corporation, Northanpton, MA 0.060, USA).

## 3. Results

### 3.1. Dynamic Change of Physicochemical Indicators of Water and Soil

**Figure 1:**
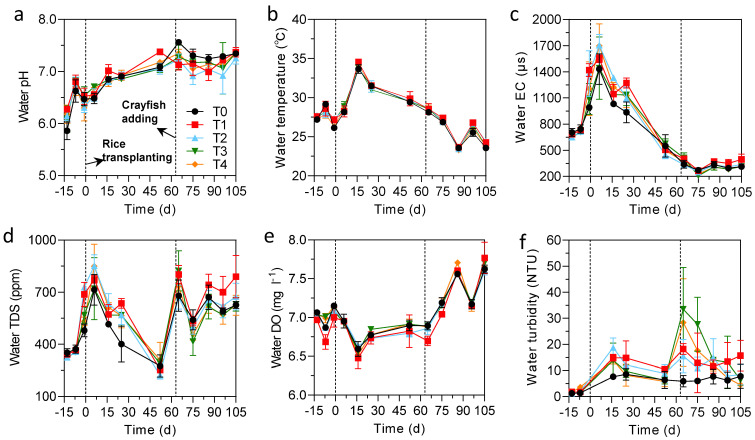
Dynamic changes of physicochemical indicators of water. (**a**) water pH; (**b**) water temperature; (**c**) water EC; (**d**) water TDS; (**e**) water DO; (**f**) water turbidity. The X-axis represents the experimental schedule.

The values of all eight physicochemical indicators: water pH, water TDS, water DO, water turbidity, and soil pH after crayfish addition were significantly or extremely significantly higher than those before crayfish addition, while water temperature and water EC after crayfish addition were significantly lower than those before crayfish addition (*p* < 0.0001). The change in soil ORP before and after crayfish addition was not significant (*p* > 0.05) (Figure S2). Dynamic changes in the physicochemical indicators of water and soil are shown in Figure 1. The pH of the different treatments was relatively similar; it increased slowly after rice transplanting and remained stable (~7.2) after adding crayfish. The water temperature reached a peak (34.0 °C) on the 16th day after rice transplanting, then gradually decreased to 28.5 °C before thr crayfish addition, then it continued to decrease to 23.9 °C, with small fluctuations until the end of the experiment. Water EC showed a similar dynamic change rule to water temperature; however, its variability among the different treatments was slightly larger. The change in water TDS was extremely volatile, it rapidly increased to 782.0 ppm after rice transplanting, and then decreased to (269.5 ppm) on the 52nd day; it continued to rapidly increase to 742.3 ppm after crayfish addition but decreased to 489.4 ppm on the 75th day, and then stayed relatively stable until the end of the experiment. Water dissolved oxygen (DO) decreased from 7.0 mg L^−1^ on the 15th day to 6.6 mg L^−1^ on the 16th day after transplanting, and then it increased slightly and remained stable at 6.9 mg L^−1^. After crayfish was added, water DO continued to increase and reached 7.7 mg L^−1^ before the end of the experiment. The variability of water turbidity among different treatments was large; it was generally stable after the transplanting of rice, but increased rapidly and then decreased after crayfish addition, and stabilized at 8.5 NTU at the end of the experiment. Soil pH was consistent with water pH, but soil pH was generally slightly lower than water pH (Figure S3). The soil redox potential (ORP) fluctuated greatly and gradually increased to 217.1 mv on the 52nd day after the transplanting of rice; it decreased to 100.1 mv after crayfish addition, and then gradually increased and stabilized at 159.3 mv until the end of the experiment.

### 3.2. Dynamic Change of Cd Concentration of Water and Soil

**Figure 2:**
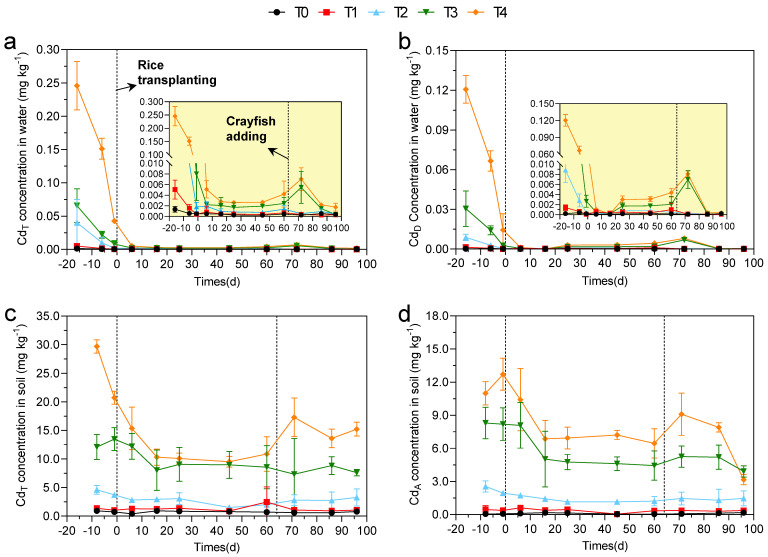
Dynamic changes of Cd in water and soil: (**a**) total Cd (Cd_T_) in water; (**b**) dissolved Cd (Cd_D_) in water; (**c**) total Cd (Cd_T_) in soil; (**d**) available Cd (Cd_A_) in soil. The X-axis represents the experimental schedule.

The Cd_T_ and Cd_D_ concentrations in water are shown in Figure 2a,b. For the majority of samples, the Cd_T_ concentration in the T0 treatment was low from the beginning to the end of the experiment, and that of the T4 treatment was significantly higher than that of the other treatments (*p* < 0.05). The Cd_T_ concentrations in the T1, T2, T3, and T4 treatments exhibited a similar trend, which was high at the beginning, then dropped dramatically from −16th to 6th d and kept stable at a relatively low level before the crayfish were added. Due to the bioturbation of crayfish, Cd_T_ concentrations of T3 and T4 treatments increased slightly after crayfish addition, whereas those of T1 and T2 were still at a relatively low level. The Cd_T_ concentrations of all five treatments were kept at a low level until the end of the experiment. The Cd_D_ concentration in water showed an extremely similar trend to that of the Cd_T_ concentration in water (Figure 2b).

Figure 2c,d shows the dynamic changes of soil Cd_T_ and Cd_A_ concentrations. Overall, soil Cd_T_ concentration in T3 and T4 treatments was at a high level, while other treatments were at a low level. Within 10 days before transplanting, the Cd_T_ concentration in the soil of each treatment showed a downward trend (except for the slight increase in the T3 treatment), which was mainly due to the Cd migration from the soil to the water. After the rice transplanting, the Cd_T_ concentration in the soil of each treatment continued to decrease, and remained stable on the 16th day after transplanting. It remained stable until crayfish was added to the pool. After the crayfish addition, the Cd_T_ concentration of soil in the T2 and T4 treatments increased slightly, while the other treatments did not change much, and then each treatment remained basically stable until the end of the experiment. For Cd_A_, the Cd_A_ concentration of T3 and T4 treatments was at a high level from 3.2 mg kg^−1^ to 12.7 mg kg^−1^, while that of T0, T1, and T2 treatments was at a low level from 0.03 mg kg^−1^ to 2.5 mg kg^−1^. The Cd_A_ concentration in the soil showed a similar dynamic trend to Cd_T_.

### 3.3. Cd intake from Rice and Crayfish Consumption

**Figure 3:**
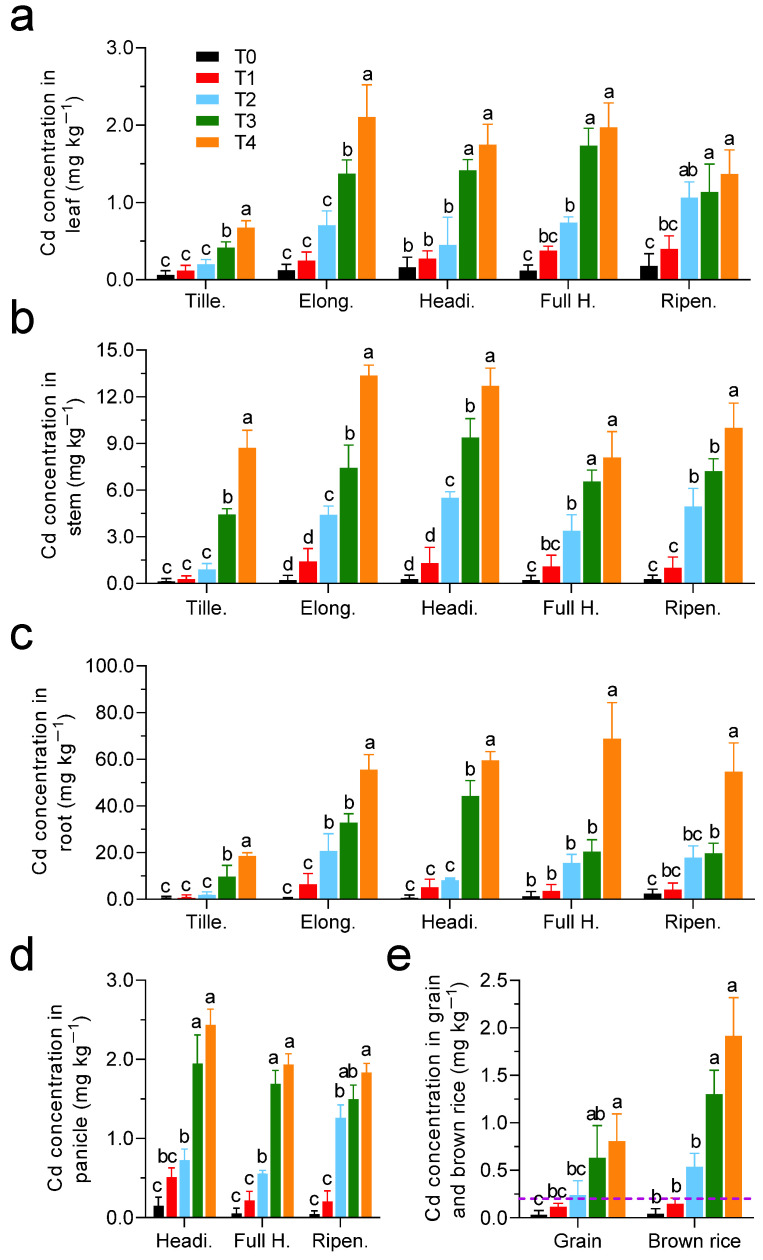
The dynamic changes of Cd concentration in rice tissues of different treatments: (**a**) leaf; (**b**) stem; (**c**) root; (**d**) panicle; (**e**) grain and brown rice. The purple line (0.2 mg kg^−1^) represents limit standard of Cd in brown rice of China according to the GB 2762-2017. Different lowercase letters represent significant levels of Cd content among different treatments (*p* < 0.05).

Among the five treatments, Cd concentration in roots was the highest, followed by stems and leaves, and those in panicles and grains were the lowest (Figure 3). With an increase in soil Cd concentration, the Cd concentration in rice tissues in each treatment increased. Figure 3 shows the dynamic changes in Cd concentration in various rice tissues during different growth periods, and those in leaves, stems, and roots were similar, at relatively low levels at the tillering stage, and gradually increased and remained stable from the elongation stage to the ripening stage. The Cd concentration in the panicles was highest in the heading stage and gradually decreased at the full heading and ripening stages. The Cd concentrations of each treatment in brown rice (0.06 ± 0.03 to 1.93 ± 0.32 mg kg^−1^) were higher than those in grain (0.05 ± 0.03 to 0.82 ± 0.22 mg kg^−1^), and the Cd concentrations of T1 and T2 treatments were lower than the limit standard of China (0.2 mg kg^−1^).

**Figure 4:**
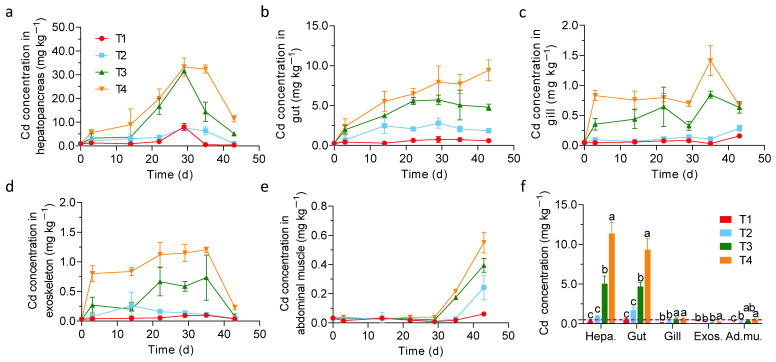
The dynamic changes of Cd concentration in crayfish organs of different treatments: (**a**) hepatopancreas; (**b**) gut; (**c**) gill; (**d**) exoskeleton; (**e**) abdominal muscle; (**f**) the Cd concentration in crayfish organs of different treatments at 43 d. Different lowercase letters represent significant levels of Cd content among different treatments (*p* < 0.05). The purple line (0.5 mg kg^−1^) represents limit standard of Cd in crustaceans of China. The X-axis represents the experimental schedule.

Dynamic changes in Cd concentrations in crayfish organs under different treatments are shown in Figure 4. Generally, of all the treatments, the Cd concentration in the hepatopancreas was the highest, followed by that in the gut, and those in the gill, exoskeleton, and abdominal muscle were the lowest. Specifically, the Cd concentration in the hepatopancreas was low at the beginning, increased gradually until it reached a peak at 29 d, and then decreased rapidly until the end of the experiment (Figure 4a). The Cd concentration in the gut showed an increasing trend from 0 d to 22 d, and then remained relatively stable until the end of the experiment (except that the T4 treatment continued to increase slowly) (Figure 4b). The Cd concentration in gill of T1 and T2 treatments remained at a low level from beginning to the end of the experiment, while those of T3 and T4 treatments varied greatly and peaked at 35 d and then decreased to around 0.66 mg kg^−1^ at 42 d (Figure 4c). The Cd concentration in the exoskeleton initially increased, then remained relatively stable, and then decreased rapidly on the 35th day (Figure 4d). The Cd concentration in the abdominal muscle was low from the beginning to the 29th day, and then increased sharply until the end of the experiment (Figure 4e). The difference in Cd concentration in crayfish organs of different treatments at 43 d reached a significant level (*p* < 0.05), especially between high (T3 and T4) and low Cd treatments (T1 and T2) for the hepatopancreas and gut (Figure 4f). The Cd concentration in the abdominal muscle of T1, T2, and T3 treatments was lower than that of the limit standard (0.5 mg kg^−1^) of crustaceans of China except for T4 treatment.

### 3.4. Soil Safety Threshold for Food Production in RCC

**Figure 5:**
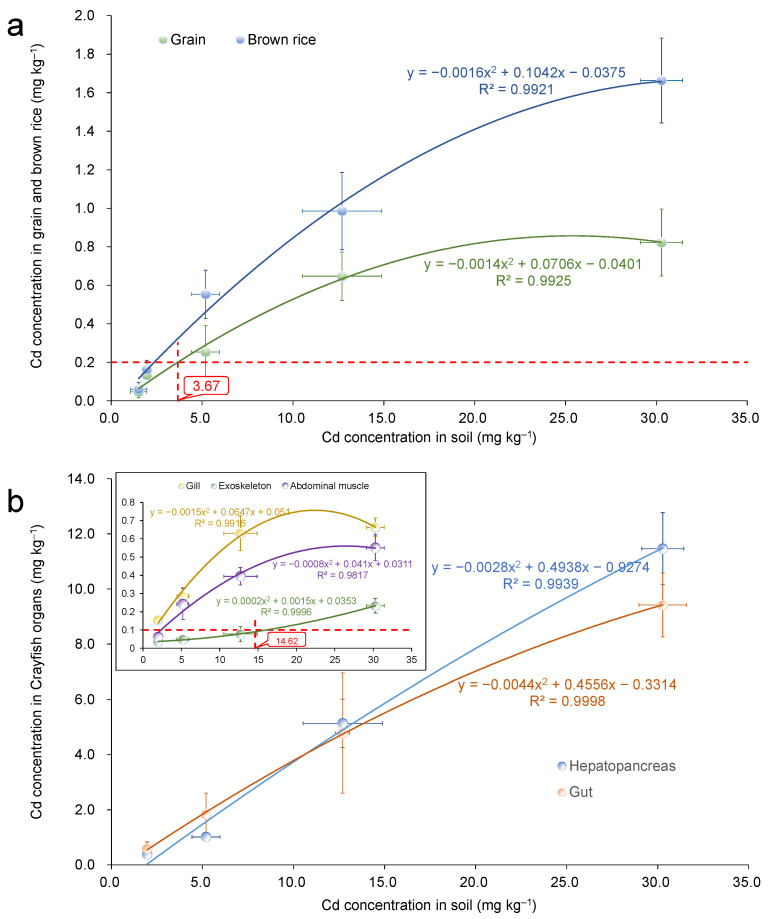
The Cd concentration in rice tissues and crayfish organs of different treatments. The red horizontal dotted line represents the limit standard of Cd in brown rice of China (0.2 mg kg^−1^) in (**a**); thus, the safety threshold of soil Cd for brown rice in the RCC was calculated to be 3.67 mg kg^−1^. The red horizontal dotted line represents the limit standard of Cd in crustaceans of China (0.1 mg kg^−1^) in (**b**); thus, the safety threshold of soil Cd for crayfish muscle in the RCC was calculated to be 14.62 mg kg^−1^.

The Cd concentration of brown rice was positively correlated with the soil Cd concentration and could be fitted by a binomial model (y = −0.0016x^2^ + 0.1042x − 0.0375, r^2^ = 0.9921) (Figure 5a). The Cd concentration in grains was also positively correlated with the soil Cd concentration and could be fitted by a binomial model (y = −0.0014x^2^ + 0.0706x – 0.0401, r^2^ = 0.9925). Thus, based on this fitted model, the soil Cd safety threshold was determined to be 3.67 mg kg^−1^, according to the maximum limit of Cd in grains in China.

The Cd concentration in each organ of crayfish increased with increasing soil Cd concentration, and all of them could be fitted by binomial models (Figure 5b). Specifically, the Cd concentration in the abdominal muscles of crayfish was positively correlated with the soil Cd concentration, and the fitting equation was y = 0.0002x^2^ + 0.0015x + 0.0353 (r^2^ = 0.9996). Based on this fitted model, the soil Cd safety threshold was determined to be 14.62 mg kg^−1^, according to the maximum limit of Cd in crustacean aquatic products in China. In conclusion, the soil Cd safety threshold for food safety production under the rice-crayfish coculture system is 3.67 mg kg^−1^.

### 3.5. The Relationship between Cd Distribution in Rice Tissues and Crayfish Organs and Environmental Factors

**Figure 6:**
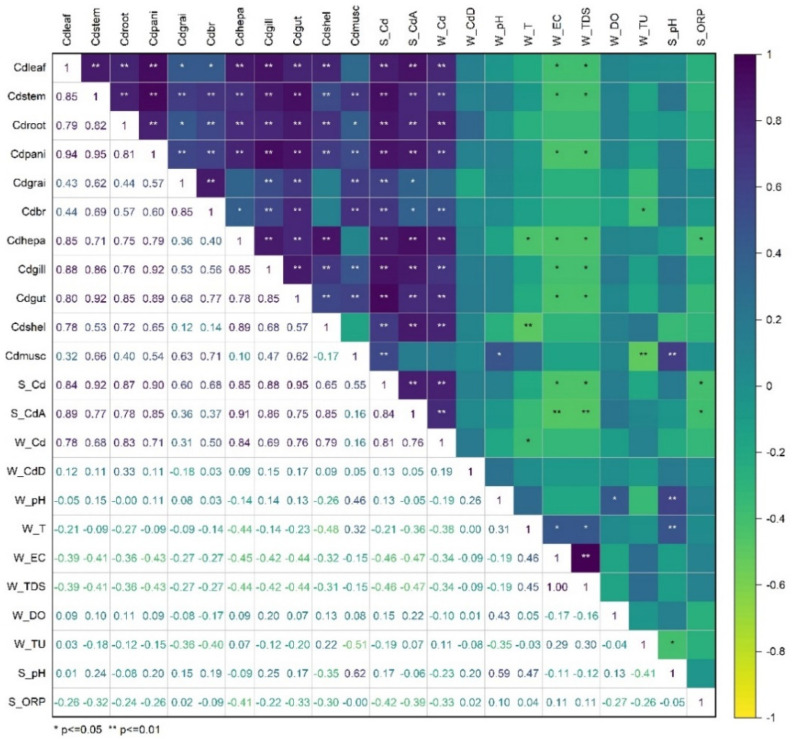
The relationship between Cd distribution in rice tissues and crayfish organs and environmental factors. The Cd_leaf_, Cd_stem_, Cd_root_, Cd_pani_, Cd_grai_, and Cd_br_ represent Cd concentration in leaf, stem, root, panicle, grain, and brown rice of rice. The Cd_hepa_, Cd_gill_, Cd_gut_, Cd_shel_, and Cd_musc_ represent Cd concentration in hepatopancreas, gill, gut, exoskeleton, and abdominal muscle of crayfish. S and W represent soil and water, respectively. Cd_A_ and Cd_D_ represent available Cd and dissolved Cd, respectively.

Correlation analysis was performed to identify the environmental factors affecting Cd distribution in various tissues and organs of rice and crayfish (Figure 6). The Cd concentration in each tissue of rice showed significant (*p* < 0.05) or extremely significant (*p* < 0.01) positive correlation with soil total Cd and soil available Cd content, and the correlation coefficient was between 0.36 and 0.92; it was extremely significant with total Cd in water (*p* < 0.01), and the correlation coefficient was between 0.31 and 0.83, but there was no significant correlation with dissolved Cd in water. The Cd concentrations in the rice leaves, stems, and roots were significantly negatively correlated with the EC and TDS of water (*p* < 0.05). In addition, there was a significant (*p* < 0.05) or extremely significant (*p* < 0.01) positive correlation between Cd concentrations in various tissues of rice, and the correlation coefficient was between 0.43 and 0.95. There was also a significant (*p* < 0.05) or extremely significant (*p* < 0.01) positive correlation between the Cd concentration in various tissues of rice and the Cd concentration in most organs of crayfish.

Except for the insignificant correlation between Cd in abdominal muscles of crayfish and soil available Cd, the Cd concentration in each organ of crayfish was positively correlated with the soil total Cd and available Cd, with correlation coefficients ranging from 0.55 to 0.95 (*p* < 0.01). The total Cd concentration in the water was significantly positively correlated with the Cd concentration in the hepatopancreas, gut, gill, and exoskeleton of crayfish (*p* < 0.01), whereas the correlation with the Cd concentration in the abdominal muscle was not significant. There was no significant correlation between the Cd concentration in each crayfish organ and the dissolved Cd concentration in the water. The Cd concentration in the hepatopancreas, gill, and gut of crayfish was significantly negatively correlated with the EC and TDS of water (*p* < 0.05), and the correlation with other physicochemical indicators of water and soil was not significant.

## 4. Discussion

Using the established Cd-contaminated rice-crayfish coculture system, this study quantitatively analyzed the migration law of Cd at the water-soil-rice-crayfish interface and the distribution characteristics of Cd in various tissues and organs of rice and crayfish, respectively. To the best of our knowledge, this is the first study to understand the impact of Cd pollution on food safety in a rice-crayfish coculture system. Our study determined the soil Cd safety threshold for food production under the rice-crayfish coculture system, which is helpful for the safe management of agricultural products in the main distribution area of the rice-crayfish coculture system, with the safety risk of Cd contamination.

In the rice-crayfish co-culture system, the physicochemical properties of soil and water were strongly affected by crayfish bioturbation. For example, in this study, the water turbidity increased from 10.8 NTU to 13.09 NTU after crayfish was added (Figure S2). Therefore, the photosynthesis of plants in water is weakened, which may reduce the amount of DO in the water. However, the amount of DO in the water after crayfish addition increased significantly compared with that of before crayfish addition, which may be mainly because crayfish preyed on plankton, which reduced the DO consumed in the water and finally manifested as an increase in DO (Figure 1). However, the results of this study are contrary to those of previous studies, in which the DO of the water decreased significantly after the addition of fish (*p* < 0.05), which may be related to the consumption of DO by the fish itself and the degradation of bait and feces [27]. After rice transplanting, the Cd concentration in soil and water decreased rapidly and was gradually transferred to various tissues of rice. Overall, the trends are as follows: root > stem > leaf ≈ panicle > grain > brown rice, which is consistent with previous research results [32]. The jointing and heading stages are the key periods for the rapid accumulation of Cd in the aboveground tissues of rice, and the Cd concentration in the aboveground tissues of rice in this period was relatively higher than that in other periods. Many factors affect the absorption of Cd by rice, such as rice variety, irrigation regime, soil Cd concentration, and soil physicochemical properties, which brings challenges to the comparison between different studies [33,34]. In this study, correlation analysis showed that total Cd in soil and total Cd in water were positively correlated with Cd concentration in various rice tissues. Compared with total Cd, soil-available Cd was more easily absorbed by rice, and there was a significant positive correlation between it and Cd concentration in various rice tissues (*p* < 0.05). In addition, in the rice-crayfish coculture, since crayfish require deep water irrigation, the soil was in a flooded state, and the soil ORP also decreased significantly compared with that before transplanting (Figure 1). Previous studies have shown that when the soil is under reducing conditions, the content of available iron and manganese in the roots of rice increases, which competitively inhibits the entry of Cd into rice [35]. Therefore, compared with the traditional rice monoculture, the rice-crayfish co-culture system may help to reduce the accumulation of Cd in rice.

The absorption of Cd by different organs of crayfish has been reported; however, previous research has mainly focused on crayfish aquaculture. In this study, the order of Cd concentration in each organ of crayfish was hepatopancreas > gut > gill ≈ exoskeleton > abdominal muscle, which was consistent with previous research results [23,36]. The hepatopancreas, as the body’s metabolism and detoxification tissue for exogenous substances, has the highest degree of contact with exogenous toxins and a longer duration of action; therefore, the concentration of Cd is the highest in the hepatopancreas [37]. Many studies have shown that metallothionein (MT) is mainly related to the detoxification of heavy metals (such as Cd and Hg), and the entry of heavy metals into the body causes the expression of MT, which then combines with heavy metal ions to reduce tissue damage [38]. Crayfish are omnivores and in addition to crayfish feed, they usually eat plant roots or fallen leaves from underwater plants, and sometimes algae and small aquatic insects. Therefore, the higher gut Cd concentration may be due to the high Cd concentration in residual food in the gut of crayfish. In this study, the Cd concentration in the abdominal muscle was the lowest, and Cd was gradually enriched in the abdominal muscle after the crayfish was kept into the paddy field for 30 days, reaching the maximum value at the end of the experiment. Correlation analysis showed that soil total Cd, soil available Cd, and total Cd concentration significantly affected the distribution of Cd in the hepatopancreas, gut, gill, and exoskeleton, while Cd concentration in the abdominal muscle was mainly related to total soil Cd.

The identification of soil safety thresholds is affected by many factors, such as rice variety, soil physicochemical properties, research assumptions, and the simulation models applied [39]. Previous studies determined the safe threshold of soil Cd for rice monoculture systems using different prediction models. For example, Cheng et al. (2014) [40] used linear regression coupled with the Monte Carlo model method to determine the safety threshold of Cd in paddy field soils in the Hangjiahu Plain. The results suggested that when the expected probability of rice Cd reaching the standard was 70%, 80%, and 90%, the safe thresholds of soil Cd were 1.2 mg kg^−1^, 1.0 mg kg^−1^, and 0.8 mg kg^−1^, respectively. Zheng et al. (2019) [41] showed that, the safety thresholds of Cd in typical acid and neutral paddy soils of Jiangsu Province based on species sensitivity distribution (SSD) were 0.78 and 1.17 mg kg^−1^, respectively. In this study, a parabolic fitting equation was used to determine the soil Cd safety threshold for food production in a rice-crayfish coculture system, which was 3.67 and 14.62 mg kg^−1^ for rice and crayfish, respectively. These results not only showed that rice is more susceptible to Cd pollution than crayfish, but also showed that the rice safety risk of rice-crayfish coculture is relatively low compared to that of rice monoculture.

## 5. Conclusions

In this study, the dynamic migration characteristics of Cd in a rice-crayfish co-culture system were investigated. In the rice-crayfish coculture, the jointing and heading stages were critical periods for the rapid enrichment of Cd in the aboveground tissues of rice, and Cd gradually accumulated in the abdominal muscles of crayfish after 30 days of aquaculture. The concentrations of soil total Cd, soil available Cd, and water total Cd significantly affected the Cd concentration in each tissue of rice and each organ of crayfish. The bioturbation of crayfish had a significant impact on the physicochemical indicators of water and soil (except for soil ORP), and the difference before and after crayfish addition was significant (*p* < 0.05) or extremely significant (*p* < 0.01). The safety threshold of soil Cd for food safety production under the rice-crayfish coculture system was 3.67 mg kg^−1^.

## Data Availability

Data is contained within the article or supplementary material.

## Supplementary Materials

The following supporting information can be downloaded at: https://www.mdpi.com/article/10.3390/foods11233828/s1, Figure S1: The location of the study area; Figure S2: Physicochemical parameters of water and soil; Figure S3: Dynamic changes of physicochemical indicators in soil; Table S1: Agricultural management records and sampling arrangements.
