# Supplementary material for: Determination of Soil Cadmium Safety Thresholds for Food Production in a Rice-Crayfish Coculture System
Source: Foods. 2022 Nov 27;11(23):3828. doi: 10.3390/foods11233828 (PMC9740835; doi:10.3390/foods11233828)
Supplement: Supplementary file 1 [file foods-11-03828-s001.zip › foods-1993039-supplementary.pdf]

Supplementary Materials

Determination of Soil Cadmium Safety Thresholds for Food Production in a Rice-Crayfish Coculture System

Hui Gao [1,2,3,*], Xiang Peng [3], Linxiu Dai [3], Jingyong Li [3], Qian Yang [3], Zhi Dou [1,2,3] and Qiang Xu [1,2,3]

[1] Jiangsu Key Laboratory of Crop Genetics and Physiology, Jiangsu Key Laboratory of Crop Cultivation and Physiology, Agricultural College of Yangzhou University, Yangzhou 225009, China

[2] Jiangsu Co-Innovation Center for Modern Production Technology of Grain Crops, Yangzhou University, Yangzhou 225009, China

[3] Research Institute of Rice Industrial Engineering Technology of Yangzhou University, Yangzhou 225009, China*

Correspondence: gaohui@yzu.edu.cn

Table S1. Agricultural management records and sampling arrangements.

Date	Experimental schedule	Agricultural management	Sampling arrangement			
			Water	Soil	Rice	Crayfish
2021/5/28	-29	Soil and Cd adding				
2021/6/10	-16		√			
2021/6/13	-13	In situ determination				
2021/6/18	-8			√		
2021/6/19	-7	In situ determination				
2021/6/20	-6		√			
2021/6/25	-1	In situ determination	√	√		
2021/6/26	0	Rice transplanting				
2021/7/2	6	In situ determination	√	√		
2021/7/11	15	Counting of tillering				
2021/7/12	16	In situ determination	√	√		
2021/7/18	22	Counting of tillering				
2021/7/21	25	In situ determination	√	√		
2021/7/24	28	Counting of tillering				
2021/7/30	34	Counting of tillering				
2021/7/31	35				√	
2021/8/10	45		√	√		
2021/8/17	52	In situ determination				
2021/8/18	53	Counting of tillering				
2021/8/24	59				√	
2021/8/25	60	Counting of tillering	√	√		
2021/8/29	64	Crayfish adding				√
2021/8/30	65	In situ determination				
2021/9/2	68	Counting of tillering			√	
2021/9/5	71			√		
2021/9/6	72		√			
2021/9/9	75	Counting of tillering, in situ determination				√
2021/9/17	83					√
2021/9/19	85				√	
2021/9/20	86	In situ determination	√	√		
2021/9/24	90					√
2021/9/30	96	In situ determination	√	√		√
2021/10/8	104					√
2021/10/9	105	In situ determination				
2021/10/14	110				√	
2021/10/26	122	Harvest of crayfish				
2021/10/29	125	Harvest of rice				

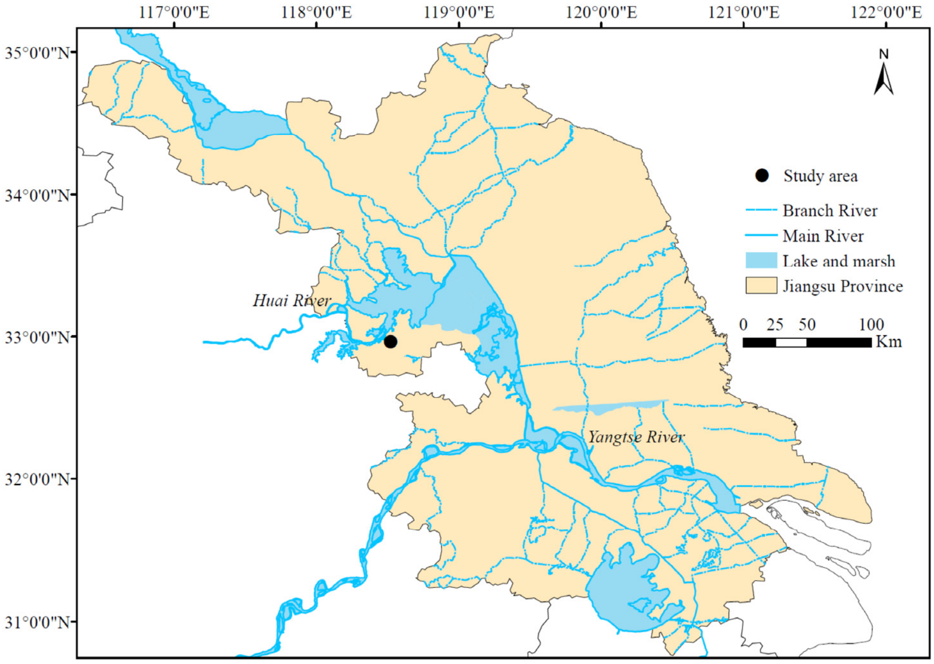

Figure S1. The location of the study area.

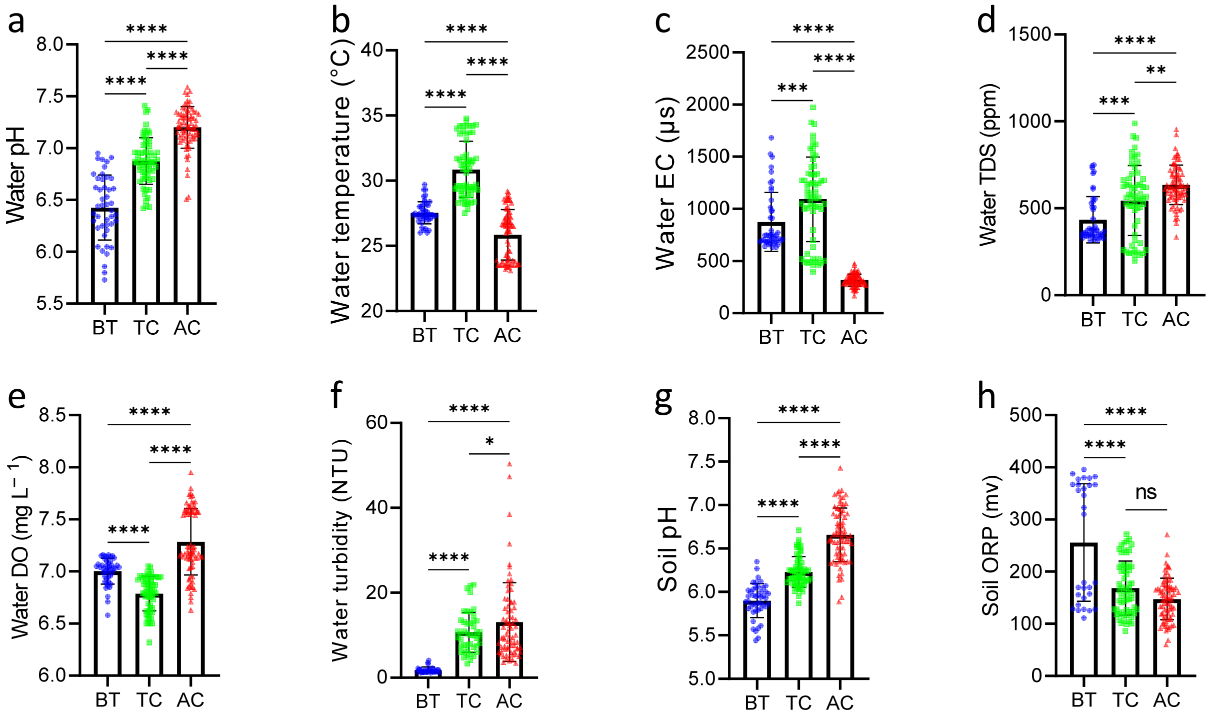

Figure S2. Physicochemical parameters of water and soil. BT, before rice transplanting; TC, from rice transplanting to crayfish adding; AC, after crayfish adding. (a) water pH; (b) water temperature; (c) water EC; (d) water TDS; (e) water DO; (f) water turbidity; (g) soil pH; (h) soil ORP. *, **, ***, and **** indicates significant differences at the p = 0.05, p = 0.01, p = 0.001, and p = 0.0001 levels (Tukey's multiple comparisons test), respectively. ns indicates not significant.

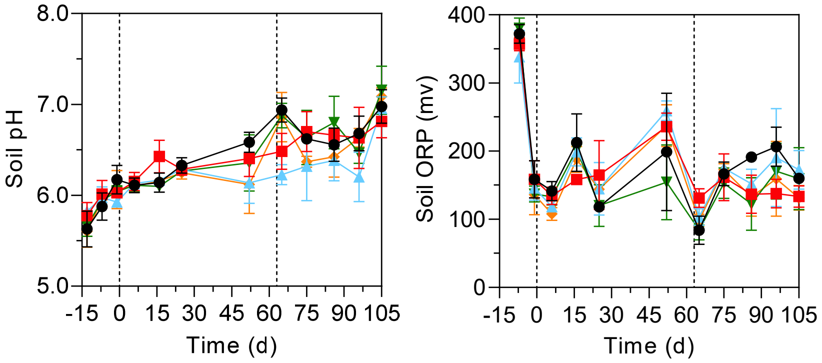

Figure S3. Dynamic changes of physicochemical indicators in soil.
